# Comment on “Misunderstanding the Match: Do Students Create Rank Lists Based on True Preferences?”

**DOI:** 10.5811/westjem.2021.4.52194

**Published:** 2021-07-20

**Authors:** Nicholas J. Connors, David M. French

**Affiliations:** HCA Healthcare/Mercer University School of Medicine, Emergency Medicine Residency Program, Charleston, South Carolina

We would like to thank the authors for exploring students’ understanding of the National Residency Match Program (NRMP) algorithm,^1^ as it is both complex and potentially confusing. In their paper, the authors make significant value judgments about what should and should not affect an applicant’s rank list. They make assumptions about what an optimal match would be for applicants and assert that a program’s opinion of an applicant is not a reason to change a preference for one residency over another. An applicant’s perceived competitiveness based on program reputation alone should not dissuade them from ranking highly a very competitive program, as the NRMP algorithm prioritizes applicants’ preferences over those of programs. However, if an applicant has some evidence that a certain program thinks especially highly of them, we believe that bit of data may suggest how a program views their fit with the residency. Programs should be cautious when alerting applicants about their relative rank list positions as applicants may interpret that as a guarantee. Ranking an applicant highly may not necessarily mean they are guaranteed to match, but rather in a position to match based on data from previous match years.

The benefits of mentorship during training are well described.^2^ Even more than mentorship, though, having a champion, one who can support and promote a resident during their training and into their post-GME career, is a rare and invaluable asset to any trainee. Making a connection with program faculty during an interview based on a shared background, professional interests, or personal goals may be the first signs of a future mentor or champion. Of course, each applicant who enters a residency should be supported by the program director (PD), associate and assistant PD, and program staff, but if there is already an indication that there is a special connection or rapport with others, this may be apparent to the applicant and may be worth ranking a particular program higher than another.

We believe the converse is also true. If a program is ranking an applicant low there may be many reasons, including a strong applicant pool, differences in weightings of the written application and interview, or potentially a poor fit based on the interview. The NRMP algorithm does favor the student; so if all other aspects of that residency program are ideal for the applicant, they may still rank a program highly even after hearing they would be ranked low by the program. Acknowledging that it would be an aberrancy for an applicant to know for certain that they will be ranked low, this information may indicate that the residency program feels the fit is not ideal, and there may be programs perceived as less competitive that might be a better fit and, ultimately, a better match for that applicant.

Applicants must assess programs based on many characteristics. Each applicant will determine their own personal algorithm for weighting each of these assessments. Factors that have been important to applicants include location and reputation, but knowing that a program will rank an applicant highly may well shine a light on the “goodness of fit,” which is described as the second most important factor in how applicants rank programs.^3^ Becoming aware of how a program rates the applicant may provide a sense of that “goodness of fit” and may be a worthwhile criteria to impel a reordering of an applicant’s rank list.
